# C10ORF10/DEPP, a transcriptional target of FOXO3, regulates ROS-sensitivity in human neuroblastoma

**DOI:** 10.1186/1476-4598-13-224

**Published:** 2014-09-28

**Authors:** Stefan Salcher, Judith Hagenbuchner, Kathrin Geiger, Maximilian A Seiter, Johannes Rainer, Reinhard Kofler, Martin Hermann, Ursula Kiechl-Kohlendorfer, Michael J Ausserlechner, Petra Obexer

**Affiliations:** Department of Pediatrics I, Medical University Innsbruck, Anichstraße 35, 6020 Innsbruck, Austria; Department of Pediatrics II, Medical University Innsbruck, Anichstraße 35, 6020 Innsbruck, Austria; Division of Molecular Pathophysiology, Medical University Innsbruck, Innrain, 80-82 6020 Innsbruck Austria; Tyrolean Cancer Research Institute, Innrain 66, 6020 Innsbruck, Austria; Department of Anesthesiology and Critical Care Medicine, Medical University Innsbruck, Anichstraße 35, 6020 Innsbruck, Austria

**Keywords:** FOXO3, DEPP, Peroxisomes, Reactive oxygen species

## Abstract

**Background:**

FOXO transcription factors control cellular levels of reactive oxygen species (ROS) which critically contribute to cell survival and cell death in neuroblastoma. In the present study we investigated the regulation of C10orf10/DEPP by the transcription factor FOXO3. As a physiological function of C10orf10/DEPP has not been described so far we analyzed its effects on cellular ROS detoxification and death sensitization in human neuroblastoma cells.

**Methods:**

The effect of DEPP on cellular ROS was measured by catalase activity assay and live cell fluorescence microscopy using the ROS-sensitive dye reduced MitoTracker Red CM-H2XROS. The cellular localization of DEPP was determined by confocal microscopy of EYFP-tagged DEPP, fluorescent peroxisomal- and mitochondrial probes and co-immunoprecipitation of the PEX7 receptor.

**Results:**

We report for the first time that DEPP regulates ROS detoxification and localizes to peroxisomes and mitochondria in neuroblastoma cells. FOXO3-mediated apoptosis involves a biphasic ROS accumulation. Knockdown of DEPP prevented the primary and secondary ROS wave during FOXO3 activation and attenuated FOXO3- and H_2_O_2_-induced apoptosis. Conditional overexpression of DEPP elevates cellular ROS levels and sensitizes to H_2_O_2_ and etoposide-induced cell death. In neuronal cells, cellular ROS are mainly detoxified in peroxisomes by the enzyme CAT/catalase. As DEPP contains a peroxisomal-targeting-signal-type-2 (PTS2) sequence at its N-terminus that allows binding to the PEX7 receptor and import into peroxisomes, we analyzed the effect of DEPP on cellular detoxification by measuring enzyme activity of catalase. Catalase activity was reduced in DEPP-overexpressing cells and significantly increased in DEPP-knockdown cells. DEPP directly interacts with the PEX7 receptor and localizes to the peroxisomal compartment. In parallel, the expression of the transcription factor peroxisome proliferator-activated receptor gamma (PPARG), a critical regulator of catalase enzyme activity, was strongly upregulated in DEPP-knockdown cells.

**Conclusion:**

The combined data indicate that in neuroblastoma DEPP localizes to peroxisomes and mitochondria and impairs cellular ROS detoxification, which sensitizes tumor cells to ROS-induced cell death.

**Electronic supplementary material:**

The online version of this article (doi:10.1186/1476-4598-13-224) contains supplementary material, which is available to authorized users.

## Background

Decidual Protein induced by Progesterone (DEPP) was originally identified as fasting- and progesterone-induced gene. DEPP is regulated via progesterone in endometrial stromal cells and via insulin levels in adipose tissue and liver and is induced in malignant glioma cells in response to hypoxic stress. It is highly expressed in various tissues including placenta, ovary, kidney, white adipose and liver. The amino acid sequence of DEPP contains a peroxisomal-targeting-signal-type-2 (PTS2) sequence, which suggests that DEPP may be imported into peroxisomes. However, the physiological functions of DEPP remain largely unknown [1–3].

In neuroblastoma cells an increased activity of the phosphatidylinositol-3 kinase (PI3K) protein kinase B (PKB/AKT) pathway was reported that contributes to therapy resistance and is associated with phosphorylation and functional inactivation of the transcription factor FOXO3 [4, 5]. Phosphorylation of FOXO3 by PKB at distinct amino acids leads to its association with 14-3-3 proteins, resulting in export from the nucleus and as a consequence thereof loss of target gene regulation in neuroblastoma cells [6]. Phosphorylation of FOXO3 by stress-induced kinases such as mammalian Ste20-like kinase (MST1) or c-Jun N-terminal kinase (JNK) in turn stimulates nuclear entry, leading to the activation or repression of target genes that affect growth, cell cycle progression, apoptosis and longevity [7–9].

In neuroblastoma cells, FOXO3 regulates cellular apoptosis by activating the two BH3-only proteins PMAIP1/Noxa and BCL2L11/Bim [5] and sensitizes these tumor cells to chemotherapy-induced cell death by repressing the IAP-family member BIRC5/Survivin [10]. Recently we also demonstrated that DNA-damaging agents activate FOXO3 and thereby cause reactive oxygen species (ROS) formation at the mitochondria due to uncoupling of mitochondrial respiration through the BH3-only protein Bim [11]. ROS are generated as side products of mitochondrial respiration [12]. Under normal conditions, low amounts of ROS are mainly detoxified in peroxisomes by the enzyme CAT/catalase [13], in the mitochondria by superoxide dismutase (SOD2) as well as by members of the sestrin family [6, 9] and by peroxiredoxins which are located in diverse organelles dependent on the cell type [14]. Catalase converts hydrogen peroxide to water and oxygen [15] and is also described as a direct transcriptional target of FOXO transcription factors in various cell types [16, 17]. It is not regulated in neuroblastoma cells [11]. High levels of cellular ROS cause oxidation of proteins, nucleic acids and intracellular membranes thereby impairing cell growth, cellular survival and proliferation [18, 19].

The transcription factor peroxisome proliferator-activated receptor gamma (PPARG) was described to be critical for the regulation of ROS steady state levels, as it directly influences the expression of several ROS-detoxifying enzymes, among them also catalase [20]. The PPARG promoter is repressed by FOXO1 in adipocytes - on the other hand PPARG can also repress the transcriptional activity of FOXO1 [21, 22].

Also beta-Catenin, which is regulated via the Wnt-pathway [23], represses PPARG expression and interacts with both, FOXO3 and PPARG via its TCF/Lef1 binding site (reviewed in [24]). DEPP may affect Wnt-signaling and thereby PPARG expression via its Pro-Pro-Pro-Ser-Pro (PPPSP) motif that has been shown to activate the Wnt-pathway [3, 25].

In the present study we investigated the regulation of DEPP by FOXO3 in human neuroblastoma cells and addressed its effects on cellular ROS household and tumor growth.

## Results

### FOXO3 regulates DEPP expression on mRNA and protein level in human neuroblastoma cells

**Figure 1:**
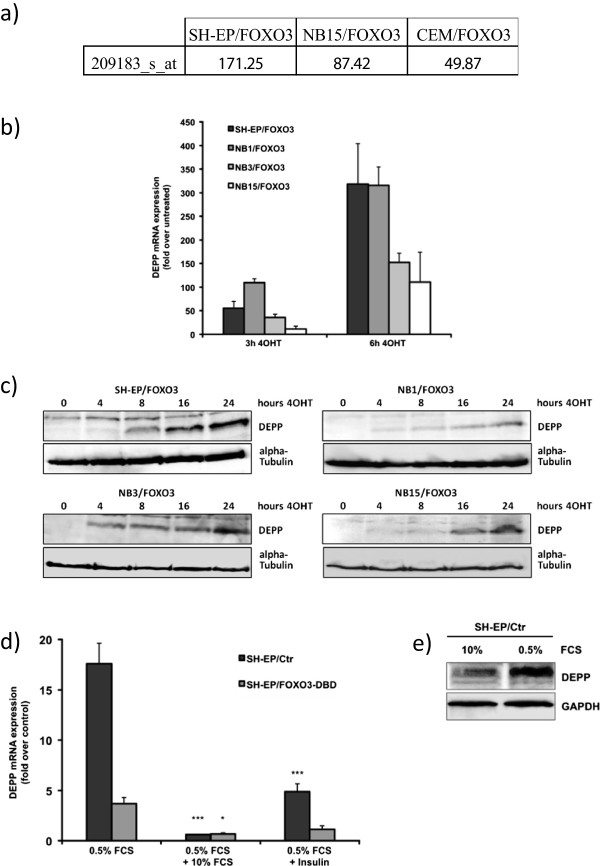
**FOXO3 induces DEPP expression on mRNA and protein level. a)** Regulation of DEPP (probe set 209183_s_at) as measured by HG-U133 Plus 2.0 microarrays. Shown are fold-increases in expression between 3 hours 4OHT treated and untreated samples. **b)** Quantitative RT-PCR of DEPP expression in SH-EP/FOXO3, NB1/FOXO3, NB3/FOXO3 and NB15/FOXO3 cells treated for 0, 3 and 6 hours with 100 nM 4OHT. Shown are means ± s.e.m. of three independent experiments, each performed in triplicates. **c)** Immunoblot analyses of DEPP expression in SH-EP/FOXO3, NB1/FOXO3, NB3/FOXO3 and NB15/FOXO3 cells treated with 100 nM 4OHT for 0, 4, 8, 16 and 24 hours. Alpha-Tubulin served as loading control. **d)** Quantitative RT-PCR of DEPP expression in SH-EP/Ctr and SH-EP/FOXO3-DBD cells, which were cultured in 0.5% FCS for 24 hours and then transferred into standard media (10% FCS) or treated with 10 nM insulin for another six hours. Shown are mean values ± s.e.m of three independent experiments, each performed in triplicates; statistical analysis was done with the Student’s unpaired *t*-test, *P < 0.05; ***P < 0.01 compared to corresponding controls. **e)** Immunoblot analysis of DEPP expression in SH-EP/Ctr cells, which were cultured in 0.5% FCS for 24 hours. GAPDH served as loading control.

DEPP was identified as a FOXO3-induced gene by Affymetrix gene-chip expression profiling analysis in cells which stably express a 4-hydroxy-tamoxifen-inducible (4OHT), PKB-phosphorylation-independent FOXO3(A3)ERtm transgene. After activation of FOXO3 by treatment with 100 nM 4OHT for 3 hours, DEPP expression was induced in the neuroblastoma cell lines SH-EP/FOXO3 and NB15/FOXO3 171 and 87 fold compared to untreated controls and in the leukemia cell line CEM/FOXO3 50 fold (Figure 1a). To verify the observed DEPP regulation by the transcription factor FOXO3, DEPP mRNA expression was further examined by quantitative RT-PCR in the neuroblastoma cell lines NB1/FOXO3, NB3/FOXO3, NB15/FOXO3 and SH-EP/FOXO3. Activation of the ectopic FOXO3(A3)ERtm via treatment with 4OHT led to significant induction of DEPP mRNA expression up to 300 fold in SH-EP/FOXO3 and NB1/FOXO3 cells (Figure 1b). To study the regulation of DEPP via FOXO3 at protein level, immunoblot analyses were performed. Activation of FOXO3 by 4OHT treatment resulted in a time-dependent increase of DEPP protein expression (Figure 1c). As DEPP is described to be an insulin-responsive gene [1] we performed quantitative RT-PCR analysis to investigate the effects of insulin- and growth factor signaling on DEPP expression. For this purpose, SH-EP/FOXO3 and SH-EP/FOXO3-DBD cells [11] were cultured under low serum conditions in presence or absence of insulin. The SH-EP/FOXO3-DBD cell line contains a FOXO3 DNA-binding domain (DBD) that suppresses FOXO3-induced transcription. Growth factor withdrawal (media supplemented with 0.5% FCS) led to significantly increased DEPP mRNA expression in SH-EP cells, whereas treatment with insulin for 6 hours efficiently reduced DEPP-induction by low serum. Expression of the dominant-negative FOXO3-DBD protein in SH-EP/FOXO3-DBD cells strongly attenuated DEPP regulation by growth factor withdrawal, suggesting that FOXO transcription factors are essential for DEPP-induction during serum starvation (Figure 1d). Growth factor withdrawal also induces DEPP protein expression in SH-EP cells as shown by immunoblot analysis (Figure 1e). Taken together, these results demonstrate that DEPP is a transcriptional target of FOXO3 and regulated downstream of insulin-signaling in neuroblastoma cells.

### All three FOXO binding sites of the DEPP promoter contribute to DEPP-induction by FOXO3

**Figure 2:**
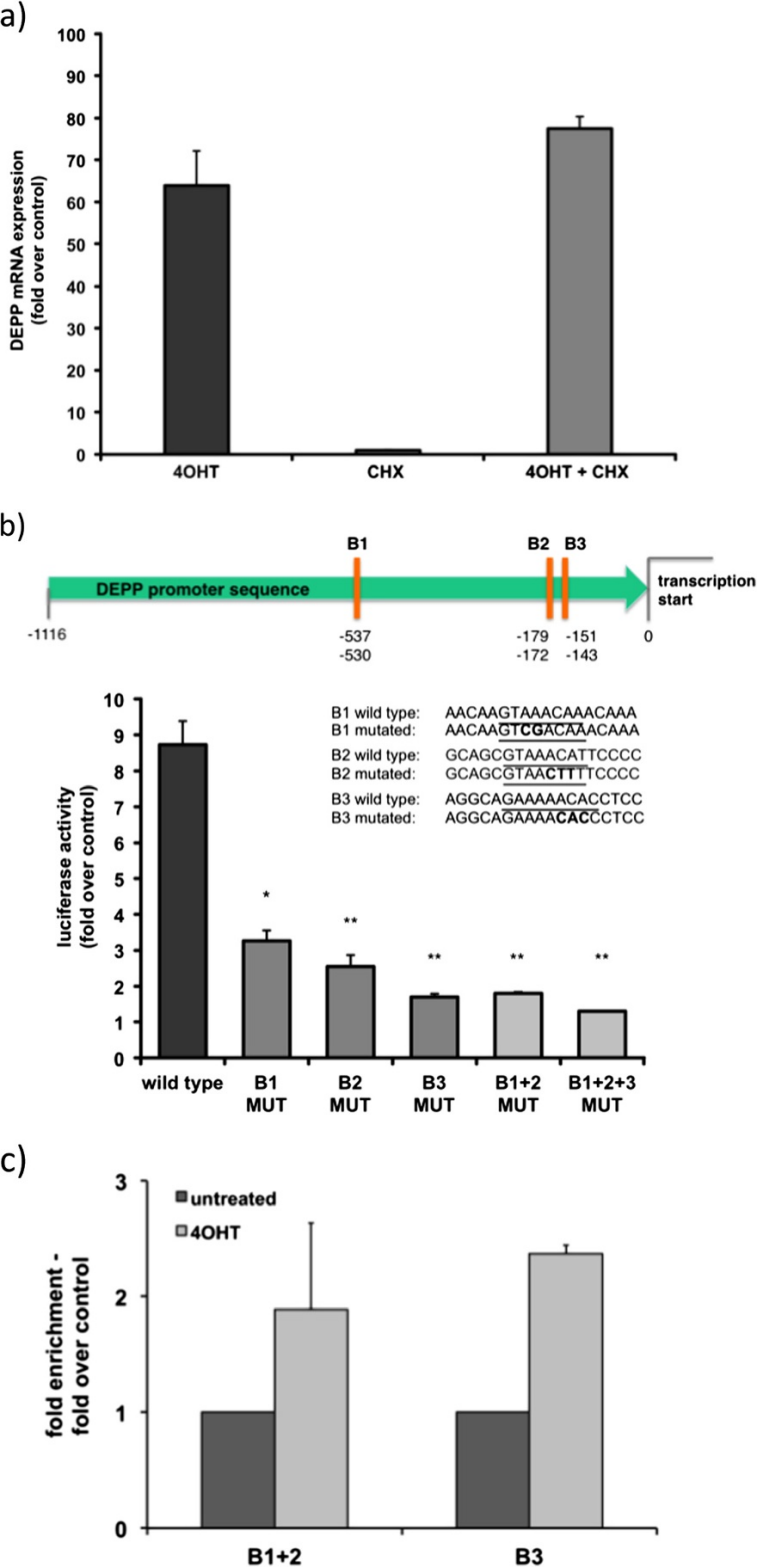
**DEPP is a direct transcriptional target of FOXO3. a)** Quantitative RT-PCR of DEPP expression in SH-EP/FOXO3 cells, treated with 75 nM 4OHT for 2 hours to activate FOXO3(A3)ERtm or a combination of 4OHT and cycloheximide (CHX; 10 μg/ml), which was used to block protein synthesis. Shown are mean values ± s.e.m. of three independent experiments, each performed in triplicates. **b)** The DEPP gene promoter (-1116 bp relative to the transcription start) contains three putative binding sites for FOXO3, which are highlighted in the schematic representation. A DEPP wild type promoter reporter plasmid and DEPP promoter reporter plasmids containing mutations of the three FOXO3-binding sites (B1, B2 and B3; indicated as bold in sequence) were transfected into SH-EP/FOXO3 cells. The cells were treated with 100 nM 4OHT for 4 hours to activate FOXO3(A3)ERtm and a luciferase-assay was performed. Direct binding of FOXO3 to the DEPP promoter leads to increased luciferase activity. The increase of the luciferase signal was calculated as fold over untreated controls. Shown are mean values ± s.e.m. of three independent experiments, each performed in duplicates; statistical analysis was done with the Student’s unpaired *t*-test, *P < 0.05; **P < 0.025 compared to corresponding controls. **c)** ChIP analysis on the interaction between FOXO3 and the FOXO3 binding sites B1 + 2 and B3 of the DEPP promoter in SH-EP/FOXO3 cells treated with 100 nM 4OHT for 3 hours. Quantification was performed by quantitative RT-PCR with specific primers for B1 + 2 and B3. Shown are mean values of two independent experiments, each performed in duplicates.

To study whether DEPP is a direct target of FOXO3 in neuroblastoma cells, quantitative RT-PCR analysis of SH-EP/FOXO3 cells treated with 75 nM 4OHT and with 10 μg/ml of the protein biosynthesis inhibitor cycloheximide (CHX) for 2 hours was performed. Treatment with CHX did not prevent the induction of DEPP after FOXO3 activation, which implies that induction of DEPP by FOXO3 does not depend on *de novo* synthesis of additional proteins, but is due to direct transcriptional regulation (Figure 2a). To further test this hypothesis, a DEPP promoter reporter luciferase assay was performed in SH-EP/FOXO3 cells using a 1116 bp genomic fragment of the promoter cloned upstream of firefly luciferase. The DEPP promoter contains three putative binding sites for FOXO3 (Figure 2b), which were mutated for this experiment. The first binding site for FOXO3 is located at -537 (B1), the second at -179 (B2), and the third at -151 (B3) relative to the start of the DEPP mRNA [26].

Activation of ectopic FOXO3(A3)ERtm increased luciferase activity approximately 9 fold (over untreated control). Single mutations of each of the three FOXO3-binding sites markedly reduced luciferase activity, indicating that each site is necessary for efficient DEPP induction. Mutation of B1 reduced the FOXO3 response to 28% of untreated cells, whereas the mutated B2 site even further attenuated the activity to 22%. Mutation of B3 exerted the strongest effect and lowered FOXO3-responsiveness of the DEPP promoter to 15% of wildtype control. This was even less than combined mutation of B1 and B2. Combined mutation of all three FOXO3-binding sites (B1 + B2 + B3 MUT) reduced luciferase activity to control level (Figure 2b). To further strengthen these findings we performed chromatin immunoprecipitation (ChIP) analysis on the FOXO3-binding sites of the DEPP promoter (Figure 2c). These experiments demonstrated that FOXO3 binds to B1 + B2 and, with highest efficiency, to the B3 consensus sequence, which is consistent with the results obtained by the luciferase promoter reporter assay in Figure 2c. The consensus sequences B1 and B2 are in close proximity, so one RT-PCR primer pair for B1 + 2 was generated.

These data demonstrate that FOXO3 activates all three consensus elements in the DEPP promoter and that all three FOXO3 binding sites are important for DEPP regulation by FOXO3 in neuroblastoma cells.

### Knockdown of DEPP reduces FOXO3-mediated apoptosis

**Figure 3:**
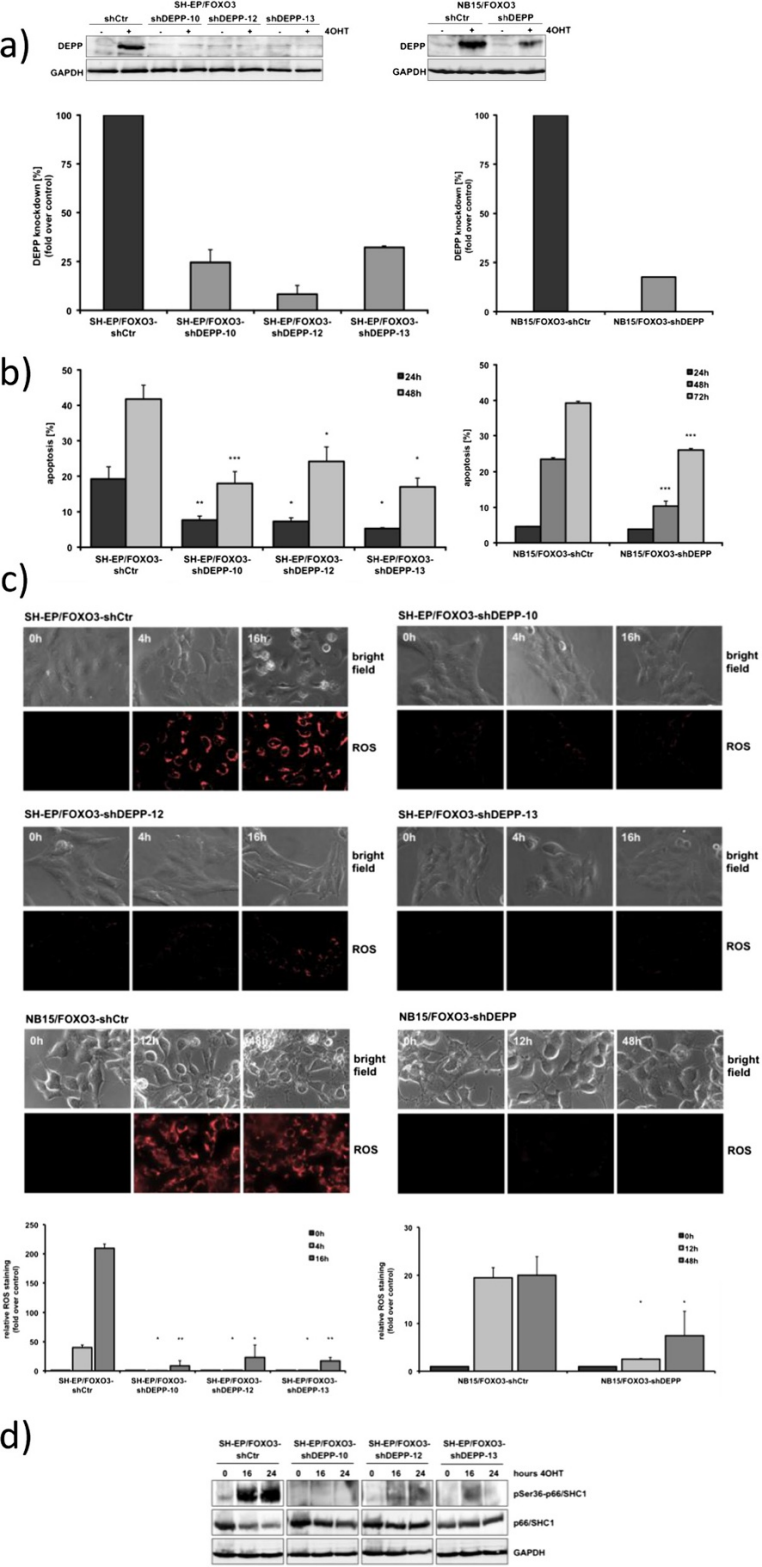
**Knockdown of DEPP reduces FOXO3-mediated apoptosis. a)** SH-EP/FOXO3 (SH-EP/FOXO3-shDEPP clone-10, -12 and -13; left panel) and NB15/FOXO3 (NB15/FOXO3-shDEPP bulk; right panel) cells were infected with vectors coding for DEPP-specific shRNA. Knockdown efficiency was verified by immunoblot (upper panel) and quantitative RT-PCR (lower panel). Cells were treated with 50 nM 4OHT to induce DEPP expression. **b)** SH-EP/FOXO-shCtr and SH-EP/FOXO3-shDEPP-clone-10, -12 and -13 as well as NB15/FOXO3-shCtr and NB15/FOXO3-shDEPP (bulk) cells were treated with 50 nM 4OHT for the indicated time points. PI-FACS analyses were performed to detect apoptotic cells. Shown is the mean ± s.e.m. of three independent experiments; statistical analysis was done with the Student’s unpaired *t*-test, *P < 0.05; **P < 0.025; ***P < 0.01 compared to corresponding controls. **c)** ROS accumulation was detected using MitoTrackerRed CM-H2XROS by live-cell imaging of SH-EP/FOXO3-shCtr and SH-EP/FOXO3-shDEPP-clone-10, -12 and -13 as well as of NB15/FOXO3-shCtr and NB15/FOXO3-shDEPP (bulk) cells treated with 50 nM 4OHT for 4 and 16 hours and for 12 and 48 hours respectively to induce FOXO3 dependent biphasic ROS accumulation. Relative ROS staining was quantified using the Axiovert200M fluorescence microscope software (Axiovision 4.6). Each panel represents the mean ± s.e.m. of 50 to 80 cells; statistical analysis was done with the Student’s unpaired *t*-test, *P < 0.05; **P < 0.025 compared to corresponding controls. **d)** Immunoblot analysis of p66/SHC1 and phosphorylated pSer36-p66/SHC1 expression in SH-EP/FOXO3-shCtr and SH-EP/FOXO3-shDEPP-clone-10, -12 and -13 cells treated with 50 nM 4OHT for the indicated time points. GAPDH served as loading control.

FOXO3 activation has been shown to induce apoptosis in neuroblastoma cells [5]. To study a possible effect of DEPP on FOXO3-mediated apoptosis, the DEPP expression was knocked down by lentiviral expression of DEPP-specific shRNA as shown in Figure 3a. Three individual clones of SH-EP/FOXO3-shDEPP (Figure 3a, left panel) and bulk-selected NB15/FOXO3-shDEPP (Figure 3a, right panel) were analyzed by immunoblot and quantitative RT-PCR analysis. Propidium iodide-(PI) FACS-analysis showed significantly reduced FOXO3-mediated apoptosis in shRNA-expressing neuroblastoma cell lines (Figure 3b).

We recently demonstrated that FOXO3-induced apoptosis is associated with and mediated by a biphasic accumulation of ROS [11]. Thus we analyzed ROS steady state levels in DEPP-knockdown cells and controls at the specific time points by live-cell imaging. Knockdown of DEPP almost completely prevented both, the primary (4 hours and 12 hours) and secondary (16 and 48 hours) ROS increase during FOXO3-activation in SH-EP/FOXO3 and NB15/FOXO3 cells, respectively (Figure 3c). In a previous study we observed that the oxidoreductase p66/SHC1 is strongly phosphorylated at Ser36 during FOXO3-induced ROS accumulation [11]. We therefore performed immunoblot analysis of p66/SHC1 and pSer36-p66/SHC1 protein in SH-EP/FOXO3-shCtr and SH-EP/FOXO3-shDEPP cells (three individual clones), which demonstrated that p66/SHC1 was not phosphorylated after FOXO3 activation in DEPP-knockdown cells (Figure 3d). This is consistent with the pronounced effect of DEPP-knockdown on FOXO3-induced ROS accumulation demonstrated by live-cell fluorescence imaging analyses (Figure 3c).

We have previously shown that BCL2L11/Bim induction [11] and BIRC5/Survivin repression [10] are essential for FOXO3-induced ROS accumulation and cell death. Knockdown of DEPP neither prevented Bim induction nor Survivin repression (data not shown), suggesting that knockdown of DEPP does not interfere with the initiation phase of ROS production but might affect cellular ROS detoxification.

**Figure 4:**
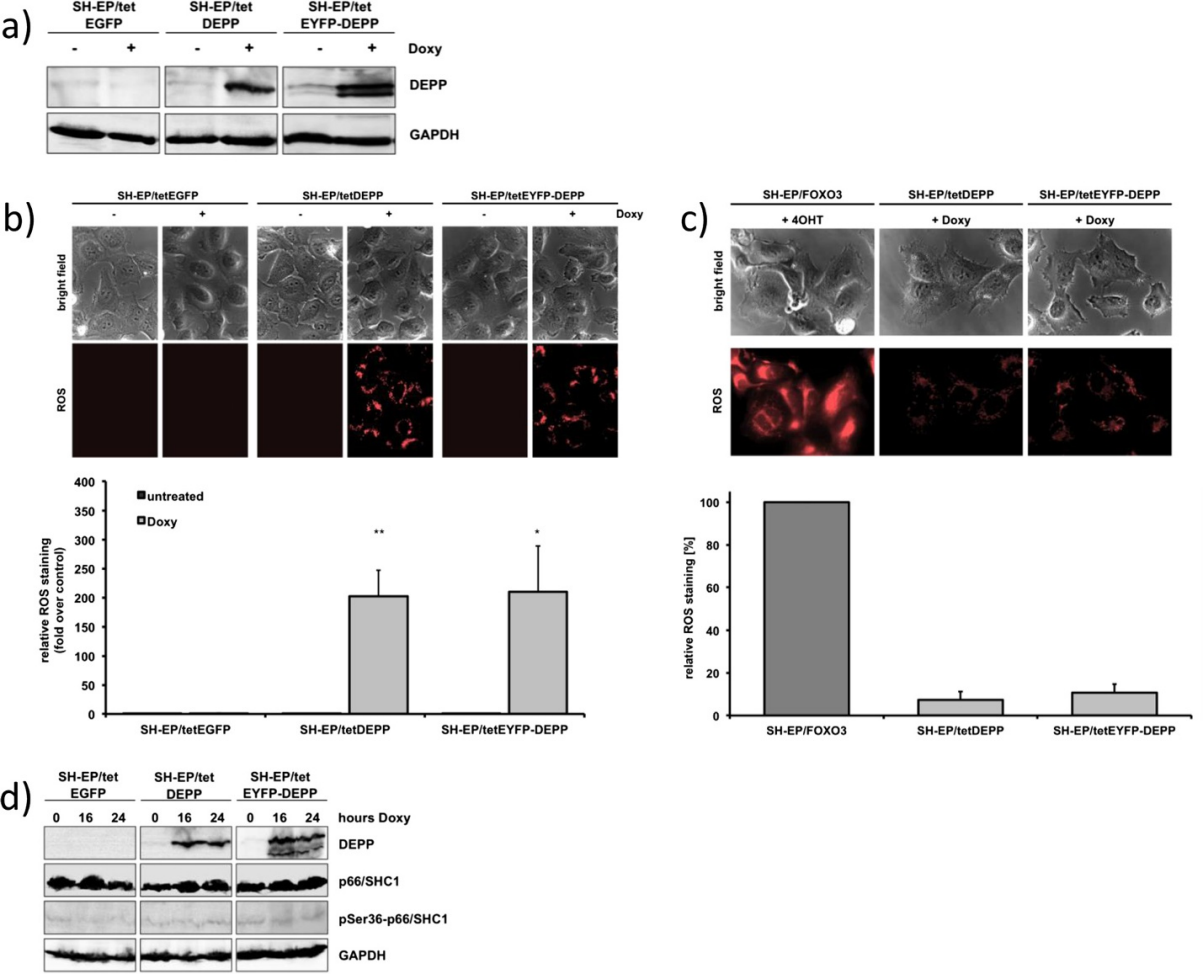
**Increased cellular ROS levels as a result of DEPP overexpression do not induce apoptosis. a)** SH-EP/tetEGFP, SH-EP/tetDEPP and SH-EP/tetEYFP-DEPP cells were treated with 200 ng/ml doxy for 24 hours to induce DEPP expression. The protein expression of DEPP was determined by immunoblot. GAPDH served as loading control. **b)** SH-EP/tetEGFP, SH-EP/tetDEPP and SH-EP/tetEYFP-DEPP cells were treated with 200 ng/ml doxy for 24 hours and ROS levels were detected by live-cell imaging using MitoTrackerRed CM-H2XROS. Relative ROS staining was quantified using the Axiovert200M fluorescence microscope software. Each panel represents the mean ± s.e.m. of 60 to 70 cells; statistical analysis was done with the Student’s unpaired *t*-test, *P < 0.05; **P < 0.025 compared to corresponding controls. **c)** SH-EP/tetDEPP and SH-EP/tetEYFP-DEPP cells were treated with 200 ng/ml doxy for 24 hours and SH-EP/FOXO3 cells with 50 nM 4OHT for 16 hours to induce FOXO3-mediated ROS accumulation. ROS levels were detected by live-cell imaging using MitoTrackerRed Red CM-H2XROS. Relative ROS staining was quantified using the Axiovert200M fluorescence microscope software. Each panel represents the mean ± s.e.m. of 10 to 20 cells. **d)** Cell lysates of SH-EP/tetEGFP, SH-EP/tetDEPP and SH-EP/tetEYFP-DEPP cells treated with 200 ng/ml doxy for 16 and 24 hours were subjected to immunoblot analyses using antibodies directed against DEPP, p66/SHC1 and phosphorylated pSer36-p66/SHC1. GAPDH served as loading control.

To directly study the effect of DEPP expression on ROS production we generated stable cell lines that conditionally express DEPP (SH-EP/tetDEPP) or EYFP-tagged DEPP (SH-EP/tetEYFP-DEPP) in a tetracycline-regulated manner (Figure 4a). DEPP overexpression on its own slightly increased cellular ROS levels (Figure 4b) but did not elevate cellular ROS to the level of FOXO3-induced ROS (Figure 4c). Furthermore, DEPP overexpression alone did not increase phosphorylation of p66/SHC1 (Figure 4d). This increase of cellular ROS by conditional DEPP expression was not sufficient to induce spontaneous apoptotic cell death as demonstrated by PI-FACS analysis of SH-EP/tetDEPP and SH-EP/tetEYFP-DEPP cells treated with doxycycline (doxy) for up to 72 hours (Additional file 1: Figure S1). This suggests that elevated DEPP expression does not constitute a death signal *per se*, but rather changes the ability of neuroblastoma cells to detoxify ROS and is therefore necessary, but not sufficient, for FOXO3-induced cell death.

### DEPP regulates the catalase enzyme activity and thereby the cellular ROS detoxification capacity

**Figure 5:**
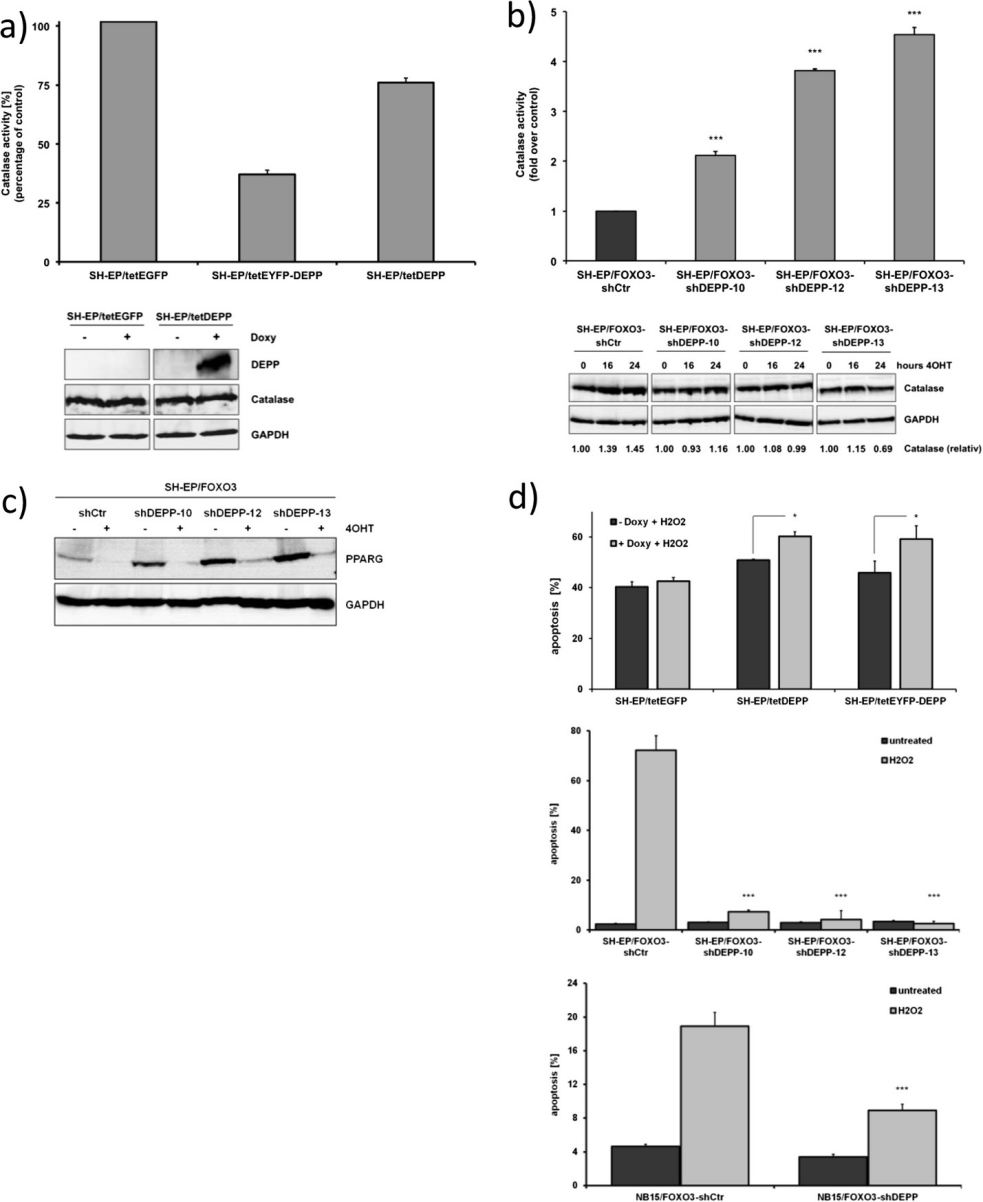
**DEPP impairs catalase activity and sensitizes to H**_**2**_ **O**_**2**_ **-induced apoptosis. a + b)** A catalase enzyme activity assay was performed in SH-EP/tetEGFP, SH-EP/tetDEPP, SH-EP/tetEYFP-DEPP cells treated with 200 ng/ml doxy **(a)** as well as of SH-EP/FOXO3-shCtr and SH-EP/FOXO3-shDEPP-clone-10, -12 and -13 cells treated with 50 nM 4OHT **(b)** for 24 hours. The catalase enzyme activity was calculated between treated and untreated cells **(a)**. Shown are mean values ± s.e.m. of three independent experiments; statistical analysis was done with the Student’s unpaired *t*-test, ***P < 0.01. Cell lysates of SH-EP/tetEGFP, and SH-EP/tetDEPP cells treated with 200 ng/ml doxy for 24 hours as well as of SH-EP/FOXO3-shCtr and SH-EP/FOXO3-shDEPP-clone-10, -12 and -13 cells treated with 50 nM 4OHT for the indicated time points were subjected to immunoblot analyses using an antibody against catalase. GAPDH served as loading control. Quantification of catalase protein expression normalized to GAPDH **(b)** was done with the ImageJ 1.48 software. **c)** Immunoblot analysis of PPARG expression in SH-EP/FOXO3-shCtr and SH-EP/FOXO3-shDEPP-clone-10, -12 and -13 cells treated with 50 nM 4OHT for 24 hours was performed. GAPDH served as loading control. **d)** SH-EP/tetEGFP, SH-EP/tetDEPP and SH-EP/tetEYFP-DEPP cells (upper panel) were pre-treated with 200 ng/ml doxy for 24 hours and then incubated with 15 μM H_2_O_2_ for one hour. SH-EP/FOXO3-shCtr and SH-EP/FOXO3-shDEPP clone-10, -12 and -13 (middle panel) and NB15/FOXO3-shCtr and NB15/FOXO3-shDEPP (bulk, lower panel) were treated with 25 μM H_2_O_2_ for 1 hour. PI-FACS analyses were performed to detect apoptotic cells. Shown are mean values ± s.e.m. of three independent experiments; statistical analysis was done with the Student’s unpaired *t*-test, *P < 0.05, ***P < 0.01.

As DEPP knockdown efficiently reduced FOXO3-induced ROS accumulation and DEPP overexpression by its own increased steady state cellular ROS we next searched for a possible explanation of this effect. Under normal conditions low amounts of hydrogen peroxide (H_2_O_2_) are mainly detoxified in the peroxisomes by the enzyme CAT/catalase [13]. Catalase was also described as a direct transcriptional target of FOXO transcription factors in various cell types [16, 17]. Protein sequence analysis suggests that DEPP contains a peroxisomal-targeting-signal-2 (PTS2) in its N-terminus that allows binding to the PEX7 receptor [27] and might mediate the import of DEPP into peroxisomes [3]. As peroxisomes play an essential role in ROS control especially in cells of neuronal origin [28] we analyzed peroxisomal function by measuring catalase enzyme activity. Catalase activity was significantly reduced in SH-EP/tetDEPP and SH-EP/tetEYFP-DEPP cells after 24 hours of DEPP overexpression compared to SH-EP/tetEGFP cells (Figure 5a). On the other hand, knockdown of DEPP in SH-EP/FOXO3-shCtr cells resulted in significantly increased catalase enzyme activity after 4OHT treatment (Figure 5b). Both, in DEPP-overexpressing and DEPP-knockdown cells, the amount of catalase protein level did not change significantly (Figure 5a,b). We only observed slightly increased catalase expression (1.45 fold compared to untreated controls) after 24 hours of FOXO3 activation in SH-EP/FOXO3-shCtr cells and no regulation in the SH-EP/FOXO3-shDEPP cell clones (Figure 5b). Next, we analyzed protein expression of PPARG, which is described to increase peroxisomal proliferation [29] and to directly regulate catalase enzyme expression and activity [24, 30]. We found protein levels of PPARG strongly upregulated in SH-EP/FOXO3-shDEPP cells compared to SH-EP/FOXO3-shCtr cells (Figure 5c), which is in line with the increased catalase enzyme activity in these cells (Figure 5b). These data suggest that cellular DEPP lowers the ability of neuroblastoma cells to cope with cellular ROS as reflected by reduced PPARG protein levels and catalase enzyme activity.

**Figure 6:**
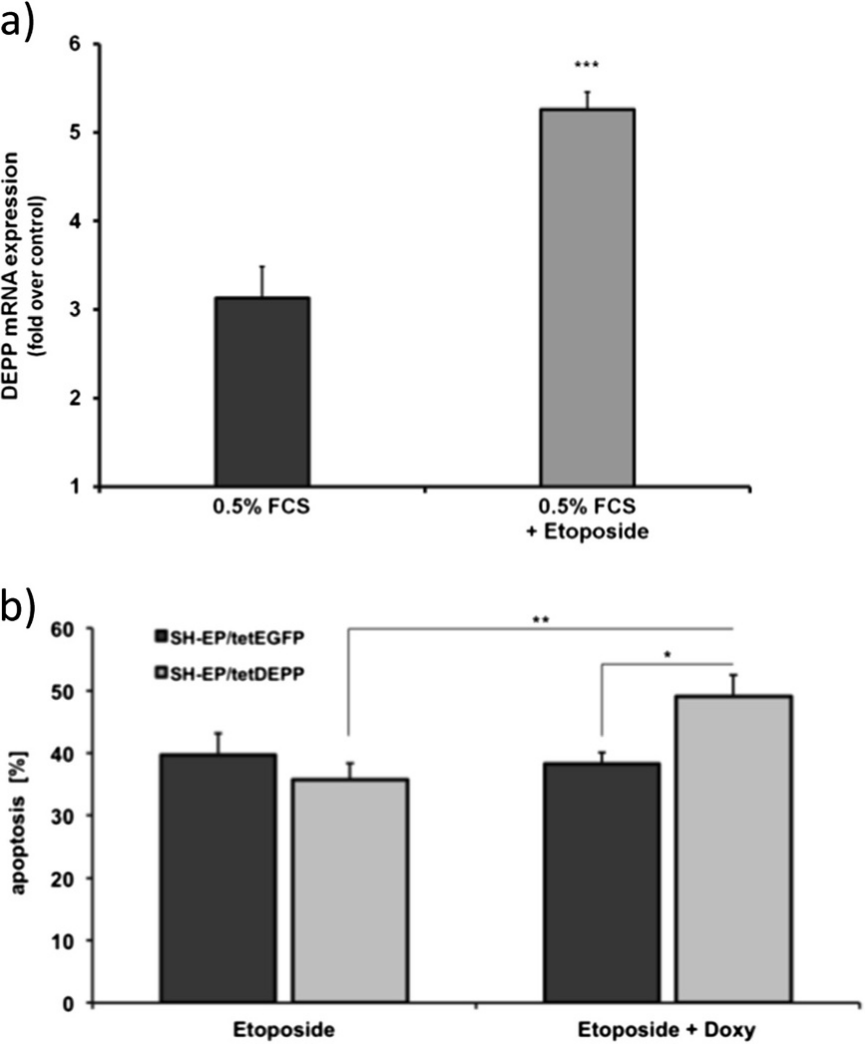
**DEPP overexpression leads to a sensitization to etoposide-induced apoptosis. a)** Real-time PCR of SH-EP cells set under serum starvation (0.5% FCS) for 24 hours and treated with 20 μg/ml etoposide for 6 hours. Shown are means ± s.e.m. of three independent experiments; statistical analysis was done with the Student’s unpaired *t*-test, ***P < 0.01. **b)** SH-EP/tetEGFP and SH-EP/tetDEPP cells were treated with 20 μg/ml etoposide alone and in combination with 200 ng/ml doxy for 48 hours. PI-FACS analyses were performed to detect apoptotic cells. Shown are mean values ± s.e.m. of three independent experiments; statistical analysis was done with the Student’s unpaired *t*-test, *P < 0.05, **P < 0.025.

To further test this hypothesis, we treated SH-EP/tetEGFP, SH-EP/tetDEPP, SH-EP/tetEYFP-DEPP, SH-EP/FOXO3-shDEPP and NB15/FOXO3-shDEPP cells with H_2_O_2_ and measured cell death by flow cytometric analysis of PI-stained nuclei. As shown in Figure 5d SH-EP/tetDEPP and SH-EP/tetEYFP-DEPP cells (upper panel) show higher H_2_O_2_-induced apoptosis, whereas SH-EP/FOXO3-shDEPP (middle panel) and NB15/FOXO3-shDEPP cells (lower panel) are significantly more resistant to H_2_O_2_ treatment than controls. Etoposide treatment of SH-EP cells activates FOXO3, leads to ROS accumulation [11] and significantly increases the expression of endogenous DEPP even under conditions of growth factor withdrawal (Figure 6a). We therefore tested the effect of the DNA-damaging agent etoposide on cells that overexpress DEPP in a tetracycline-regulated manner. As shown in Figure 6b, DEPP-overexpression led to a significant increase (P < 0.05) in etoposide-induced apoptosis. Taken together these results demonstrate that DEPP reduces the cellular ROS detoxification capacity, which in turn increases the sensitivity to H_2_O_2_-and etoposide-induced apoptosis in neuroblastoma cells.

### DEPP localizes to mitochondria and peroxisomes in neuroblastoma

**Figure 7:**
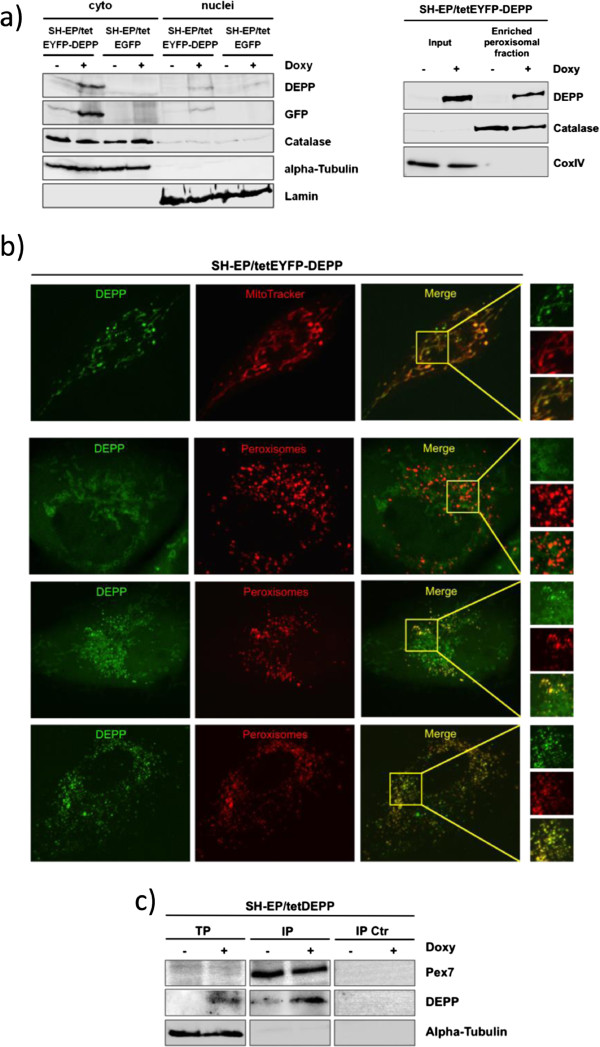
**DEPP is located in mitochondria and peroxisomes. a)** SH-EP/tetEGFP and SH-EP/tetEYFP-DEPP cells were treated with 200 ng/ml doxy for 24 hours and subjected to subcellular fractionation. The protein levels of DEPP, GFP and catalase in the cytosolic and the nuclear fraction were determined by immunoblot. Alpha-Tubulin (cytosolic) and Lamin (nuclear) were used to control the purity of the fractions (left panel). The peroxisomal fraction of SH-EP/tetEYFP-DEPP cells treated with 200 ng/ml doxy for 24 hours was separated using a peroxisome isolation kit (PEROX1, Sigma). DEPP protein expression was determined by immunoblot. Catalase (peroxisomes) and CoxIV (mitochondria) were used to control the purity of the peroxisomal fraction (right panel). **b)** SH-EP/tetEYFP-DEPP cells were grown on eight-well chambered cover glasses, treated with 200 ng/ml doxy for 24 hours and analyzed by live confocal microscopy. Mitochondria were stained using the specific mitochondrial probe MitoTrackerRed/CMXRos (30 nM). Peroxisomal staining was performed with a CellLight® Peroxisomes-RFP fusion construct. **c)** SH-EP/tetDEPP cells treated with 200 ng/ml doxy for 24 hours were subjected to CoIP analysis with a Pierce Crosslink Magnetic IP/Co-IP kit to investigate whether the DEPP protein co-immunopurifies with PEX7-cross-linked beads. The expression of PEX7 and DEPP was determined by immunoblot. Alpha-Tubulin served as loading control.

DEPP was described to reside exclusively in the nucleus of HEK293 cells [3], whereas Stepp et al. described DEPP as an unstable protein located in aggresomes in Vero cells [31]. To analyze the localization of DEPP in neuroblastoma cells we performed subcellular fractionation and immunoblot analyses of SH-EP/tetEGFP and SH-EP/tetEYFP-DEPP cells treated with 200 ng/ml doxy for 24 hours. As shown in Figure 7a (left panel) we found DEPP mainly present in the cytoplasmic and not in the nuclear fraction. Catalase, which is located in the peroxisomes and the mitochondria, was also mainly present in the cytoplasmic fraction. In a next step we treated SH-EP/tetEYFP-DEPP cells with 200 ng/ml doxy for 24 hours, isolated the peroxisomes and performed immunoblot analysis of DEPP expression (Figure 7a, right panel). By this subcellular fractionation experiment we clearly demonstrated that a significant proportion of DEPP is located in peroxisomes. To further analyze the localization of DEPP we performed live cell confocal imaging of SH-EP/tetEYFP-DEPP cells treated with 200 ng/ml doxy for 24 hours. Staining of the mitochondria with MitotrackerRed/CMXRos and labeling of peroxisomes by expression of a CellLight^®^ Peroxisomes-RFP marker protein clearly showed that the DEPP protein co-localizes with both, mitochondria and peroxisomes in neuroblastoma cells (Figure 7b). We detected DEPP in SH-EP/EYFP-DEPP cells exclusively in the mitochondria (Figure 7b, top two lines) or in the peroxisomes (Figure 7b, bottom line) and also simultaneously in both organelles (Figure 7b, third line). Therefore the live cell confocal imaging analyses indicate that the DEPP protein is present in both organelles and may translocate between mitochondria and peroxisomes, which are cooperating and cross-talking [32] and are both critical for the control of cellular ROS [15, 33, 34]. As DEPP lacks a mitochondrial targeting sequence but contains a PTS2-signal at its N-terminus we next studied whether DEPP is targeted to peroxisomes in a PTS2-dependent manner via interaction with the PEX7 receptor [27]. As shown in Figure 7c, DEPP co-immunopurifies with the peroxisomal PEX7 receptor in SH-EP/tetDEPP cells treated with 200 ng/ml doxy for 24 hours. This is in line with the subcellular fractionation assays (Figure 7a) and the live cell imaging experiments (Figure 7b) demonstrating that DEPP is present in peroxisomes.

## Discussion

In this study we demonstrate for the first time that the FOXO3-regulated gene DEPP impairs ROS detoxification via the enzyme catalase and thereby increases the effects of ROS on FOXO3-induced apoptosis in neuroblastoma cells. Using Affymetrix gene expression profiling we identified DEPP as a FOXO3-regulated gene (Figure 1a) in neuroblastoma as well as CEM-C7H2 leukemia cells and found DEPP also induced by FOXO3 at the protein level (Figure 1c). Recently it was reported that DEPP is transcriptionally regulated by FOXO3 in human endothelial cells and that FOXO3 binds to the DEPP promoter at -151 (B1) and -179 (B2) relative to the transcription start [26]. However, we found that the direct induction of DEPP by FOXO3 is critically mediated via a third binding site (B3), which is located -537 relative to the transcription start of the DEPP mRNA in neuronal cells (Figure 2b). Both, luciferase-reporter assays and ChIP analyses demonstrate that FOXO3 binds to the three FOXO-consensus sequences, among them most efficiently to the binding site B3 (Figure 2c).

DEPP expression is inhibited by insulin-growth signaling in neuroblastoma cells (Figure 1d). This is in line with studies describing downregulation of DEPP mRNA in mouse 3T3-L1 adipocytes, rat H4IIE and human HepG2 hepatoma cells as a result of insulin treatment [1, 35]. However, in neuroblastoma cells DEPP-induction by growth factor withdrawal is strongly reduced in the presence of a dominant-negative FOXO mutant suggesting that DEPP regulation by insulin/growth factor signaling almost exclusively relies on FOXO transcription factors (Figure 1d).

Currently there is little known about the physiological function of DEPP as the few studies available focused on the mechanisms of DEPP regulation. Chen and colleagues found a prominent increase of DEPP expression in endothelial cells when cultured under hypoxic conditions [26]. Similar results had been reported before in a malignant glioma cell line [36]. These results may indicate a possible role of DEPP during cellular stress response.

In this paper we demonstrate that the induction of DEPP by FOXO3 contributes to FOXO3-induced cell death as DEPP-knockdown significantly reduced FOXO3-mediated apoptosis (Figure 3b). This death-protective effect of DEPP-knockdown was associated with and possibly also mediated by a marked reduction of FOXO3-induced ROS accumulation (Figure 3c). FOXO3 causes phosphorylation at Ser-36 of p66/SHC1, which correlates with the time point of the second ROS accumulation and apoptosis induction in SH-EP/FOXO3 cells [11]. This phosphorylation of p66/SHC1 was almost completely prevented by DEPP-knockdown (Figure 3d). On the other hand, forced DEPP-overexpression in the absence of FOXO3-induction slightly increased cellular ROS levels, but importantly did not elevate cellular ROS to the level of FOXO3-induced ROS formation. This might explain why DEPP-overexpression did not lead to phosphorylation of p66/SHC1 and apoptosis induction by its own (Figure 4). These combined data suggest that in neuronal cells DEPP may act as a sensitizer for cellular ROS that affects ROS accumulation and/or detoxification.

DEPP contains a PTS2 signal sequence at its N-terminus [3] suggesting that this protein might get imported into peroxisomes, which in turn critically mediate the oxidative stress response via the enzyme catalase [15]. Catalase activity was reduced in response to DEPP overexpression and significantly increased in DEPP-knockdown cells, although catalase was not regulated on protein level (Figure 5a,b). In parallel, the expression of PPARG was strongly upregulated in DEPP-knockdown cells. This transcription factor is critical for the control of cellular ROS levels as it directly regulates several different ROS-detoxifying enzymes, including catalase [20]. PPARG acts as a regulator of peroxisomal proliferation and its upregulation may explain the marked resistance of DEPP-knockdown cells towards H_2_O_2_ (Figure 5d). This repression by FOXO3 (Figure 5c) is consistent with earlier studies that demonstrated direct transcriptional repression of the PPARG promoter by FOXO1, which recognizes the same consensus sequence as FOXO3 [21, 22]. PPARG on the other hand can also repress FOXO transcriptional activity. Beta-Catenin, which interacts with both, FOXO and PPARG via its TCF/Lef1 binding site, represses PPARG expression (reviewed in [24]). We found beta-Catenin expression up-regulated as a result of DEPP overexpression (Additional file 2: Figure S2). One explanation for this phenomenon could be that DEPP stimulates the Wnt-pathway via its PPPSP motif [3, 23, 25]. As beta-Catenin mediates repression of PPARG, this could explain the observed increase in PPARG expression in DEPP-knockdown cells, which in turn increases ROS resistance and further shuts down FOXO3 transcriptional activity. When ectopically expressed, FOXO3 is activated, this circuit is interrupted and PPARG is repressed (Figure 5c), which also opens an avenue for ROS-accumulation during FOXO3-induced cell death. This possible link between DEPP, beta-Catenin and PPARG protein expression will be investigated in a separate project.

Reduced catalase activity results in increased levels of intracellular H_2_O_2_ and subsequent cellular damage. Furthermore it was described that the increase in mitochondrial oxidative damage and the decrease in mitochondrial function occurs rapidly following the inhibition of peroxisomal catalase [15]. H_2_O_2_ causes oxidative damage throughout the cell and mainly impairs mitochondria, which in turn leads to further ROS accumulation. In particular H_2_O_2_, which is the species of ROS that accumulates upon catalase inhibition, freely diffuses across biological membranes, including aquaporin channels present in the mitochondrial membranes [37]. Consistent with the changes in catalase activity, we found that DEPP overexpression increased ROS levels and H_2_O_2_-induced apoptosis, whereas DEPP-knockdown cells were more resistant to H_2_O_2_ treatment (Figure 5d).

In transient overexpression studies using HEK293T cells DEPP was reported to localize to the nucleus [3] and not to peroxisomes as predicted by the presence of a PTS2 signal in the N-terminus. Using subcellular fractionation (Figure 7a) and live cell confocal imaging with fluorescent peroxisomal and mitochondrial probes (Figure 7b) we demonstrate that in neuroblastoma cells DEPP is a cytoplasmatic protein that localizes in part to peroxisomes and to mitochondrial structures. Co-immunoprecipitation analyses (Figure 7c) indicate that DEPP is targeted to peroxisomes in a PTS2-dependent manner as it interacts with the PEX7 receptor.

As these two cell organelles are the key regulators of cellular stress response and ROS generation/detoxification, DEPP might directly modulate ROS at these organelles.

The above data predict that DEPP may act as a sensitizer for all forms of apoptotic cell death that involve accumulation of ROS as a second messenger. Indeed, this is true for FOXO3-induced apoptosis, where knockdown of DEPP significantly lowered FOXO3-induced cell death (Figure 3b). DEPP overexpression on the other hand increased etoposide-induced apoptosis. Of note, etoposide also activates endogenous DEPP expression, thereby limiting the visible effect of ectopic expression on death sensitivity (Figure 6). Importantly, induction of DEPP by etoposide and death-sensitization by DEPP are in line with our previous work, which demonstrated that etoposide-induced cell death depends on the activation of FOXO3 and the subsequent induction of cellular ROS in neuronal cells [11].

## Conclusions

In neuroblastoma, FOXO3 gets activated as a result of cellular stress response, which leads to cellular ROS formation and upregulation of DEPP expression. The combined data clearly demonstrate for the first time that DEPP regulates cellular ROS levels, reduces catalase enzyme activity and may thereby support or even amplify ROS accumulation during FOXO3-induced apoptosis.

## Methods

### Cell lines, culture conditions, and reagents

The neuroblastoma cell lines STA-NB1, STA-NB3 and STA-NB15 were isolated at the St. Anna Children’s Hospital (Vienna, Austria) and are termed NB1, NB3 and NB15, respectively [38]. SH-EP cells were kindly provided by N. Gross, Lausanne, Switzerland [39]. The acute lymphoblastic leukemia cell line CEM/C7H2, a subclone of the CCRF-CEM cell line and all other cell lines were cultured in RPMI 1640 (Lonza, Basel, Switzerland) containing 10% fetal calf serum, 100 U/ml penicillin, 100 μg/ml streptomycin and 2 mM L-glutamine (Gibco BRL, Paisley, GB) at 5% CO_2_ and 37°C in saturated humidity. Phoenix^TM^ packaging cells for helper-free production of amphotropic retroviruses [40] and HEK293T packaging cells for production of lentiviruses were cultured in DMEM (Lonza, Basel, Switzerland). Cell culture was tested routinely for mycoplasma contamination using the VenorRGeM-mycoplasma detection kit (Minerva Biolabs, Germany). All reagents were purchased from Sigma-Aldrich (Vienna, Austria) unless indicated otherwise. For each experiment, mid-log-phase cultures were seeded in fresh medium.

### Retroviral and lentiviral expression vectors

The vectors pLIB-FOXO3(A3)-ER-iresNeo, pQ-tetCMV-SV40-Neo and pQ-tetCMV-EGFP-SV40-Neo have been described previously [5, 41, 42]. For conditional gene expression, the coding region of DEPP was amplified from human cDNA with primers containing appropriate restriction enzyme sites. The fragment was inserted into the MfeI and XhoI sites of the tet-regulated expression plasmid pQ-tetCMV-SV40-Neo generating the plasmid pQ-tetCMV-DEPP-SV40-Neo and into the MfeI and XhoI sites of the EYFP-containing plasmid pQ-tetCMV-EYFP-SV40-Neo (pQ-tetCMV-EYFP-DEPP-SV40-Neo). The lentiviral vectors coding for human DEPP-specific shRNA and the control vector pLKO.1 were obtained from Sigma-Aldrich (Vienna, Austria). pLIB-mycTag-FOXO3-DBD-iresPuro was constructed by inserting the FOXO3-DBD fragment from pSG5-MycTag-FOXO3-DBD [43] into the EcoR1 and Sal1 sites of the pLIB-MCS2-iresPuro plasmid [41].

### Production of retroviruses and lentiviruses for infection of neuroblastoma and leukemia cells

6 × 10^5^ Phoenix™ packaging cells were transfected with 2 μg of retroviral vectors and 1 μg of a plasmid coding for VSV-G protein using Lipofectamine2000 (Invitrogen, Carlsbad, USA). For production of lentiviruses 6.5 × 10^5^ HEK293T cells were transfected with 1.6 μg pLKO.1-shDEPP plasmids coding DEPP-specific shRNAs (Sigma-Aldrich, Vienna, Austria) and the packaging plasmid pCMV 8.91 (kindly provided by D. Trono, EPFL, Lausanne). After 48 hours the virus-containing supernatants were filtered through 0.22 μm syringe filters (Sartorius, Goettingen, Germany) and incubated with the target cells for at least 6 hours. SH-EP/FOXO3 and NB15/FOXO3 cells were infected to generate SH-EP/FOXO3-Ctr, SH-EP/FOXO3-shDEPP (clone-10, -12 and -13), NB15/FOXO3-Ctr and NB15/FOXO3-shDEPP (bulk-selected) cells. pQ-tetCMV-DEPP-SV40-Neo, pQ-tetCMV-EYFP-DEPP-SV40-Neo, and pQ-tetCMV-EGFP-SV40-Neo supernatants were used to generate SH-EP/tetDEPP, SH-EP/tetEYFP-DEPP and SH-EP/tetEGFP cells for doxycycline-inducible DEPP and EGFP expression using the “tet-on” system [41]. pLIB-mycTag-FOXO3-DBD-iresPuro supernatants were used to infect SH-EP cells (SH-EP/FOXO3-DBD) [11].

### Microarray data set generation and analysis

Generation of the Affymetrix microarray data set was performed at the Expression Profiling Unit of the Medical University Innsbruck according to the manufacturer’s protocols. The procedure and protocols have been described elsewhere [44]. The data analysis was performed in R (version). Raw data has been pre-processed using the GCRMA method [45]. Raw and pre-processed data has been deposited at the Gene Expression Omnibus (GEO accession number GSE53046).

### Site directed mutagenesis

A luciferase reporter plasmid containing the DEPP promoter (-1116 bp relative to the transcription start site) was purchased from Switchgear Genomics (Menlo Park, USA). The three putative FOXO3 binding sites in the DEPP promoter [26] (named B1, B2 and B3) were mutated by sited directed mutagenesis PCR using circular mutagenesis. The first site is located at -537 to -530 (B1), the second at -179 to -172 (B2), and the third at -151 to -143 (B3) relative to the transcription start (Primers for mutagenesis PCR: B1-fwd: GCTTTCGGAGGATTTGT**TTGTCGAC**TTGTTCACCAGATAT, B1-rev: ATATCTGGTGAACAA**GTCGACAA**ACAAATCCTCCGAAAGC, B2-fwd: CTGCCCTGCAGC**GTAACTTTT**CCCCAGCCTCCTAC, B2-rev: GTAGGAGGCTGGGG**AAAAGTTAC**GCTGCAGGGCAG, B3-fwd: CAGGCA**GAAAACACC**CTCCAAGCTGG, B3-rev: CCAGCTTGGAG**GGTGTTTTC**TGCCTG/ T_a_ = 58°C, 18 PCR-cycles). Promoter reporter plasmids with mutated FOXO-binding sites B1, B2, B3, B1 + B2 and B1 + B2 + B3 were used for luciferase activity analysis.

### Quantitative RT-PCR analysis

To quantify DEPP mRNA levels, we designed “real-time” RT-PCR assays, using GAPDH as reference gene. NB1/FOXO3, NB3/FOXO3, NB15/FOXO3 and SH-EP/FOXO3 cells were cultured in the presence of 100 nM 4OHT for the times indicated to activate the FOXO3(A3)ERtm transgene. Total RNA was prepared from 5 × 10^6^ cells using TRIzol^TM^ Reagent (Invitrogen, Carlsbad, USA) according to the manufacturer’s instructions. cDNA was synthesized from 1 μg of total RNA using the Revert H Minus First Strand cDNA Synthesis Kit (Thermo Scientific, Huntsville, USA). Quantitative RT-PCR was performed as described previously [11] using DEPP (forward ACTGTCCCTGCTCATCCATTCTC and reverse AGTCATCCAGGCTAGGAGAGGG) and GAPDH-specific oligonucleotides (forward TGTTCGTCATGGGTGTGAACC and reverse GCAGTGATGGCATGGACTGTG). After normalization on GAPDH expression, regulation was calculated between treated and untreated cells.

### Immunoblotting and subcellular fractionation

Immunoblot analysis and subcellular fractionation were performed as described previously [11]. The membranes were incubated with primary antibodies specific for DEPP, PEX7, GAPDH (Novus, Littleton, USA), Bim, beta-Catenin (BD Biosciences, Heidelberg, Germany), Catalase (Calbiochem, San Diego, USA), p66/SHC1, phosphorylated pSer36-p66/SHC1 (Abcam, Cambridge, UK), PPARG, Lamin, CoxIV (Cell Signaling,Danvers, USA), GFP (Sigma-Aldrich,Vienna,Austria) and alpha-Tubulin (Oncogene Research Products, Boston,USA).

After incubation with anti-mouse, anti-rat or anti-rabbit horseradish-peroxidase-conjugated secondary antibodies the blots were analyzed by enhanced chemiluminescence substrate (GE-Healthcare, Vienna, Austria) according to the manufacturer’s instructions and detected with an AutoChemiSystem (UVP, Cambridge, GB). Quantification of protein expression was done with the ImageJ 1.48 software, according to the ImageJ User Guide (http://imagej.nih.gov/ij/index.html).

### Peroxisomal separation

The peroxisomal fraction was isolated by using the peroxisome isolation kit (PEROX1; Sigma-Aldrich, Vienna, Austria), according to the instructions of the manufacturer. Briefly, 4 × 10^8^ SH-EP/tetEYFP-DEPP cells were treated with 200 ng/ml doxy for 24 hours, harvested, resuspended in peroxisome extraction buffer and homogenized in a dounce homogenizer. Next, the cells were centrifuged for 10 minutes at 1000 g. The supernatant represents the “input fraction”. After several centrifugation steps according to the manufacturer’s protocol, the pellet was collected in 1x peroxisome extraction buffer. Isolation of the peroxisomes was done on a density gradient. By a centrifugation step for 1.5 hours at 100.000 g the purified peroxisomes were separated from the mitochondria. To measure the purity of the peroxisomal fraction immunoblot analyses with catalase (peroxisomes) and CoxIV antibodies (mitochondria) were performed.

### Co-immunoprecipitation analysis (CoIP)

CoIP was performed with a Pierce Crosslink Magnetic IP/Co-IP kit (Pierce, Rockford, USA) according to the instructions of the manufacturer. Briefly, 5 μg of PEX7 antibody (Novus, Littleton, USA) were covalently cross-linked to 25 μl of A/G magnetic beads. The prepared beads as well as beads without cross-linked antibody (IP Ctr) were incubated with extracts from SH-EP/tetDEPP cells treated with 200 ng/ml doxy for 24 hours, washed to remove non-bound material and eluted in a low-pH elution buffer that dissociates bound antigen from the antibody- linked beads. The total protein (TP) and the eluates (IP and IP Ctr) were analyzed via immunoblot.

### Chromatin immunoprecipitation assay (ChIP)

ChIP was performed with a Millipore Magna ChIP Kit (Millipore, Darmstadt, Germany) according to the instructions of the manufacturer. Approximately 2 × 10^7^ SH-EP/FOXO3 cells and 20 μl of the protein G beads coupled with 5 μl of anti-FOXO3 antibody (Santa Cruz, Dallas, USA) were used for each preparation. For quantification of FOXO3 binding to the DEPP promoter quantitative real time RT-PCR was performed with primers for the binding sites B1 + B2 (forward AAAACAGCTTGGTGGGCGGG and reverse AACAAGCTTTGGGGCAGGGG) and B3 (forward CTGCTCCTAGGAGAGACACACCCTG and reverse CTGCTACGTTTGCTGTGCTTAGTGC).

### Determination of apoptosis by flow cytometry

Apoptosis was measured by staining the cells with propidium-iodide (PI) and forward/sideward scatter analysis using a CytomicsFC-500 Beckman Coulter. 2 × 10^5^ cells were harvested and incubated in 500 μl hypotonic PI solution containing 0.1% Triton X-100 for 4 to 6 hours at 4°C. Stained nuclei in the sub-G1 marker window were considered to represent apoptotic cells [46].

### Luciferase activity assay

To determine direct regulation of the DEPP-promoter by FOXO3, promoter plasmids containing the DEPP-promoter (1116 bp) and mutated variants were transiently transfected into SH-EP/FOXO3 cells using the JetPrime^®^ Reagent (Polyplus, Berkeley, USA) according to the manufacturer’s instructions. Subsequently the cells were cultured in the presence of 100 nM 4OHT for 4 hours to activate the FOXO3 transcription factor. Luciferase activity was measured with a Luciferase Assay System kit (Promega, Fitchburg, USA) according to the manufacturer’s instructions. The reactions were done in duplicates and repeated three times. Luciferase activity was calculated between treated and untreated cells.

### Live cell ROS staining

For ROS measurements, cells were grown on LabTek Chamber Slides™ (NalgeNunc International, Rochester, USA) coated with 0.1 mg/ml collagen and incubated with reduced MitoTrackerRed CM-H2XROS (Invitrogen, Carlsbad, CA, USA) for 20 minutes according to the manufacturer’s instructions (final concentration 500 nM). Images were collected with an Axiovert200M microscope equipped with filters for EYFP (exitation: BP500/20, emission: BP535/30) and RFP (excitation: BP546/12, emission: LP590) and a 63x-oil objective (Zeiss, Vienna, Austria).

### Live confocal imaging

Cells were grown on LabTek Chamber Slides™ (NalgeNunc International, Rochester, USA) coated with 0.1 mg/ml collagen and incubated for 15 minutes with 30 nM MitoTrackerRed CMX-Ros (Invitrogen, Carlsbad, USA) to stain mitochondria. Peroxisomes were labelled with the CellLight^®^ Peroxisome-RFP vector (Life Technologies, Carlsbad, USA) according to the manufacturer’s instructions. Cells were analyzed by live confocal microscopy using an inverted microscope (Zeiss Observer.Z1; Zeiss, Oberkochen, Germany) in combination with a spinning disc confocal system (UltraVIEW VoX; Perkin Elmer, Waltham, MA, USA). All images were acquired using a 63× oil immersion objective.

### Catalase assay

Catalase enzyme activity was analyzed with a Catalase Assay Kit (Abcam, Cambridge, UK). Cells were cultured in the presence of 50 nM 4OHT (SH-EP/FOXO3-shDEPP) or 200 ng/ml doxy (SH-EP/tetEGFP, SH-EP/tetDEPP, SH-EP/tetEYFP-DEPP) for the times indicated. 1 × 10^6^ cells were harvested and used for the enzyme assay according to the manufacturer’s instructions. After 30 minutes incubation time the reaction was stopped and the optical density was measured with a Benchmark Microplate Reader (BioRad Laboratories, Munich, Germany). Catalase enzyme activity was calculated between treated and untreated cells.

## Electronic supplementary material

Additional file 1: Figure S1: Elevated DEPP expression does not cause cellular apoptosis *per se*. SH-EP/tetDEPP and SH-EP/tetEYFP-DEPP cells were treated with 200 ng/ml doxy for the indicated time points. PI-FACS analyses were performed to detect apoptotic cells. Shown are means ± s.e.m. of three independent experiments. (PPTX 82 KB)

Additional file 2: Figure S2: DEPP overexpression increases beta-Catenin protein levels. SH-EP/tetEGFP, SH-EP/tetDEPP and SH-EP/tetEYFP-DEPP cells were treated with 200 ng/ml doxy for 24 hours to induce DEPP expression. The protein expression of DEPP and beta-Catenin was determined by immunoblot. GAPDH served as loading control. (PPTX 188 KB)
