# Supplementary figures and images for: C10ORF10/DEPP, a transcriptional target of FOXO3, regulates ROS-sensitivity in human neuroblastoma
Source: Mol Cancer. 2014 Sep 28;13:224. doi: 10.1186/1476-4598-13-224 (PMC4197242; doi:10.1186/1476-4598-13-224)

## Slide 1
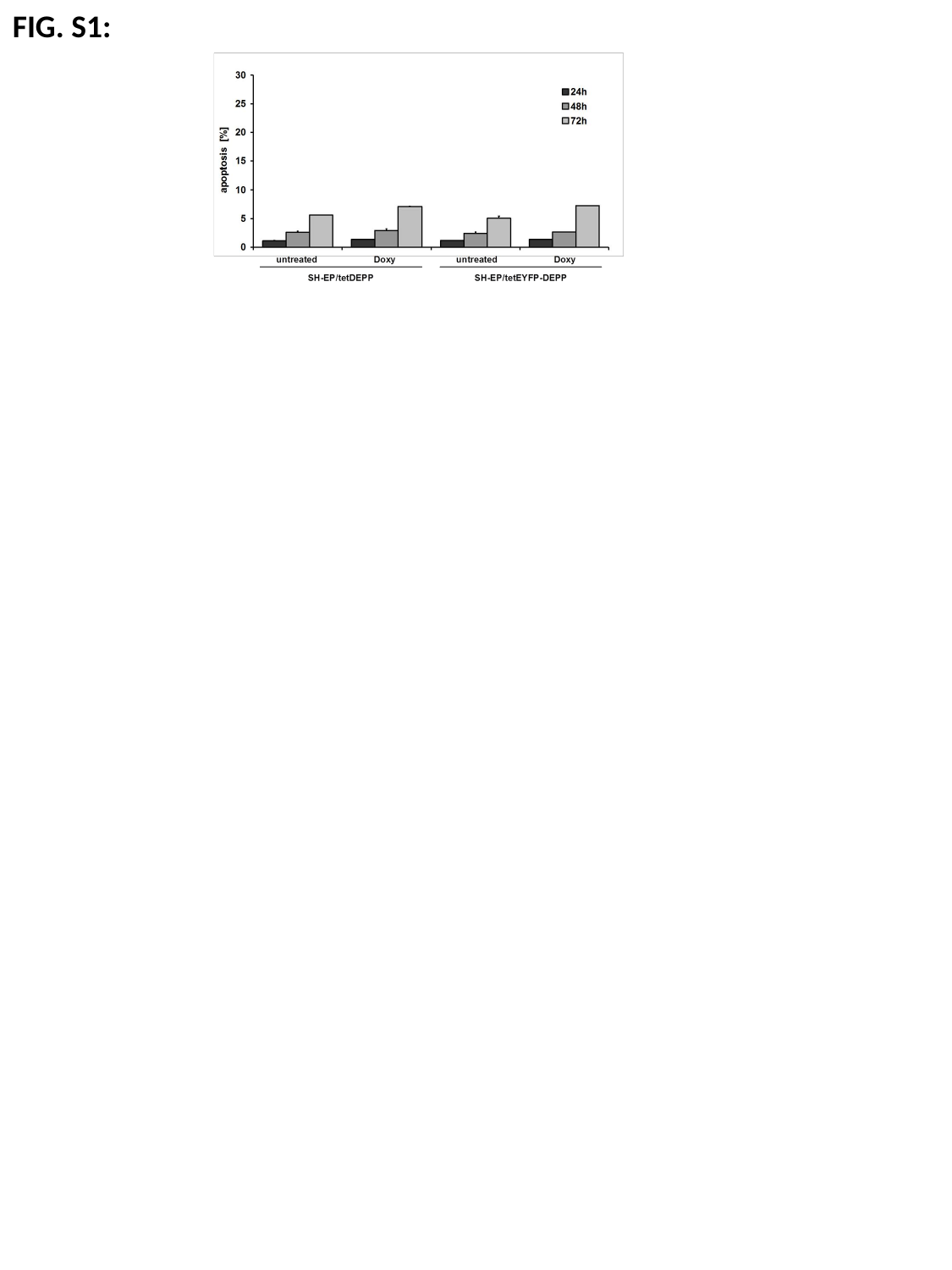

FIG. S1:

Supplement: Supplementary file 1 — Additional file 1: Figure S1: Elevated DEPP expression does not cause cellular apoptosis per se. SH-EP/tetDEPP and SH-EP/tetEYFP-DEPP cells were treated with 200 ng/ml doxy for the indicated time points. PI-FACS analyses were performed to detect apoptotic cells. Shown are means ± s.e.m. of three independent experiments. (PPTX 82 KB) [file 12943_2014_1427_MOESM1_ESM.pptx]

## Slide 1
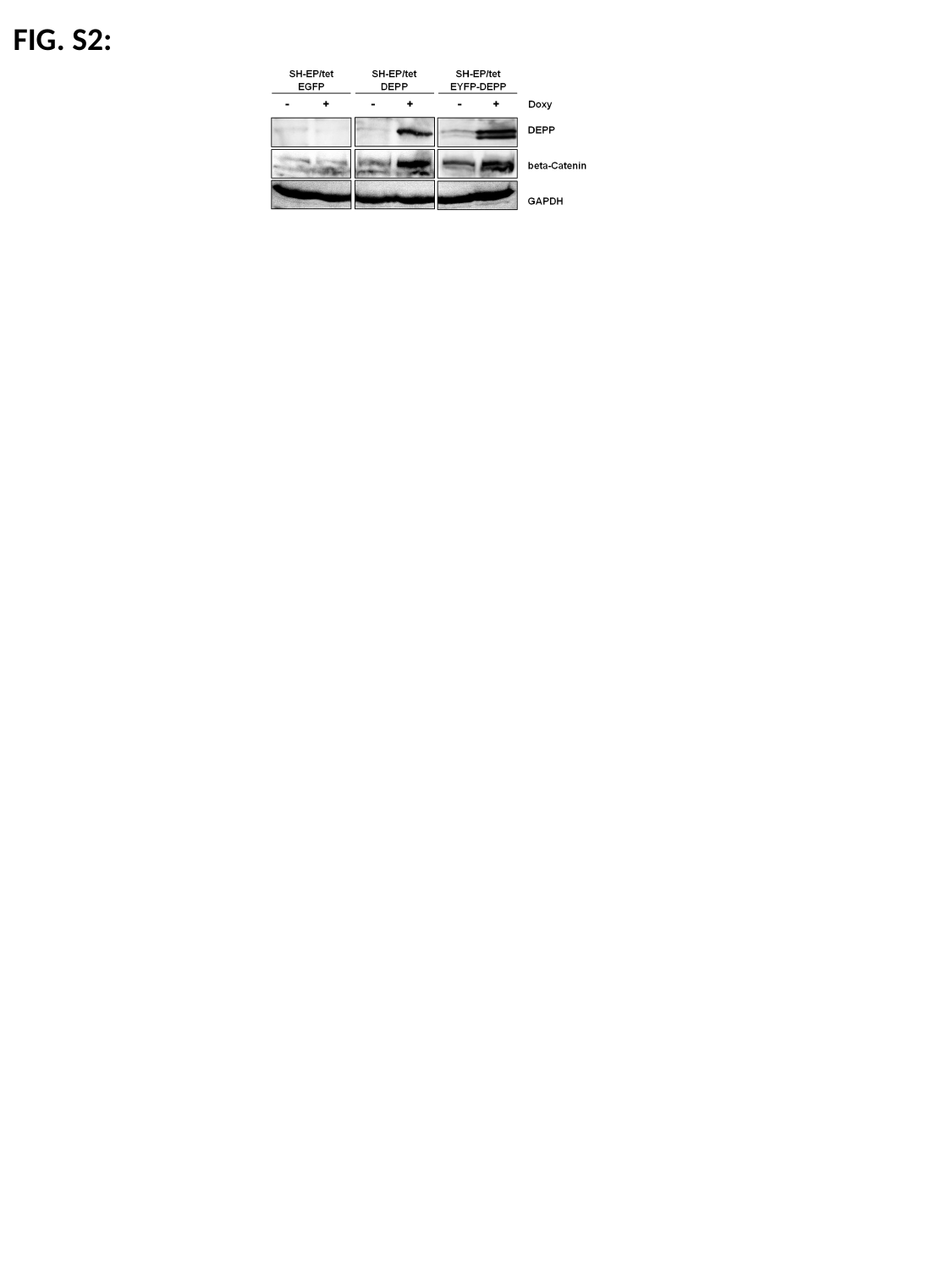

FIG. S2:

Supplement: Supplementary file 2 — Additional file 2: Figure S2: DEPP overexpression increases beta-Catenin protein levels. SH-EP/tetEGFP, SH-EP/tetDEPP and SH-EP/tetEYFP-DEPP cells were treated with 200 ng/ml doxy for 24 hours to induce DEPP expression. The protein expression of DEPP and beta-Catenin was determined by immunoblot. GAPDH served as loading control. (PPTX 188 KB) [file 12943_2014_1427_MOESM2_ESM.pptx]
